# Evaluation of sequencing reads at scale using rdeval

**DOI:** 10.1093/bioinformatics/btaf416

**Published:** 2025-07-22

**Authors:** Giulio Formenti, Bonhwang Koo, Marco Sollitto, Jennifer Balacco, Nadolina Brajuka, Richard Burhans, Erick Duarte, Alice M Giani, Kirsty McCaffrey, Jack A Medico, Eugene W Myers, Patrik Smeds, Anton Nekrutenko, Erich D Jarvis

**Affiliations:** The Vertebrate Genome Laboratory, The Rockefeller University, 1230 York Ave, New York City, NY 10065, United States; The Vertebrate Genome Laboratory, The Rockefeller University, 1230 York Ave, New York City, NY 10065, United States; The Vertebrate Genome Laboratory, The Rockefeller University, 1230 York Ave, New York City, NY 10065, United States; Department of Biology, University of Florence, Sesto Fiorentino, FI 50019, Italy; The Vertebrate Genome Laboratory, The Rockefeller University, 1230 York Ave, New York City, NY 10065, United States; The Vertebrate Genome Laboratory, The Rockefeller University, 1230 York Ave, New York City, NY 10065, United States; Department of Biochemistry and Molecular Biology, The Pennsylvania State University, 505 Wartik Laboratory, University Park, PA 16801, United States; The Vertebrate Genome Laboratory, The Rockefeller University, 1230 York Ave, New York City, NY 10065, United States; Feil Family Brain and Mind Research Institute, Weill Cornell Medicine, New York, NY 10021, United States; The Vertebrate Genome Laboratory, The Rockefeller University, 1230 York Ave, New York City, NY 10065, United States; The Vertebrate Genome Laboratory, The Rockefeller University, 1230 York Ave, New York City, NY 10065, United States; The Vertebrate Genome Laboratory, The Rockefeller University, 1230 York Ave, New York City, NY 10065, United States; Department of Biochemistry and Molecular Biology, The Pennsylvania State University, 505 Wartik Laboratory, University Park, PA 16801, United States; Department of Biochemistry and Molecular Biology, The Pennsylvania State University, 505 Wartik Laboratory, University Park, PA 16801, United States; The Vertebrate Genome Laboratory, The Rockefeller University, 1230 York Ave, New York City, NY 10065, United States

## Abstract

**Motivation:**

Large sequencing datasets are being produced and deposited into public archives at unprecedented rates. The availability of tools that can reliably and efficiently generate and store sequencing read summary statistics has become critical.

**Results:**

As part of the effort by the Vertebrate Genomes Project (VGP) to generate high-quality reference genomes at scale, we sought to address the community’s need for efficient sequence data evaluation by developing rdeval, a standalone tool to quickly compute and interactively display sequencing read metrics. Rdeval can either run on the fly or store key sequence data metrics in tiny read ‘snapshot’ files. Statistics can then be efficiently recalled from snapshots for additional processing. Rdeval can convert fa*[.gz] files to and from other popular formats including BAM and CRAM for better compression. Overall, while CRAM achieves the best compression, the gain compared to BAM is marginal, and BAM achieves the best compromise between data compression and access speed. Rdeval also generates a detailed visual report with multiple data analytics that can be exported in various formats. We showcase rdeval’s functionalities using long-read data from different sequencing platforms and species, including human. For PacBio long-read sequencing, our analysis shows dramatic improvements in both read length and quality over time, as well as the benefit of increased coverage for genome assembly, though the magnitude varies by taxa.

**Availability and implementation:**

Rdeval is implemented in C++ for data processing and in R for data visualization. Precompiled releases (Linux, MacOS, Windows) and commented source code for rdeval are available under MIT license at https://github.com/vgl-hub/rdeval. Documentation is available on ReadTheDocs (https://rdeval-documentation.readthedocs.io). Rdeval is also available in Bioconda and in Galaxy (https://usegalaxy.org). An automated test workflow ensures the consistency of software updates.

## 1 Introduction

Over the past 25 years, we have witnessed an unprecedented increase in the number of publicly available sequencing datasets (Lewin *et al.* 2022, Larivière *et al.* 2024). Data have been generated using a variety of sequencing platforms, with Illumina short reads being by far the most abundant. Since 2010, Pacific Biosciences (PacBio) and Oxford Nanopore Technologies (ONT) have revolutionized sequencing with the introduction of radically new long-read sequencing platforms (Giani *et al.* 2020), producing reads in the order of 10 s to 100 s of kb. While the base-calling accuracy of Illumina short reads had been considerably higher than that of long reads for many years, recent improvements in both PacBio and ONT are now rivaling if not exceeding Illumina read quality. In 2022, the expiration of Illumina’s short-read patents paved the way for new short-read platforms, including Singular Genomics, Ultima Genomics, MGI, and Element Biosciences’ AVITI (Pollie 2023).

Sequencing data are made publicly available by large national and international initiatives such as the Sequence Read Archive (SRA) by the US National Center for Biotechnology Information (NCBI, https://www.ncbi.nlm.nih.gov/sra/), the European Nucleotide Archive (www.ebi.ac.uk/ena/browser/home) by the European Bioinformatics Institute, the DNA Data Bank of Japan (www.ddbj.nig.ac.jp), the China National Genebank (https://db.cngb.org), or in project-related repositories such as the GenomeArk (https://www.genomeark.org) by the Vertebrate Genomes Project (VGP) (Rhie *et al.* 2021). Several of these initiatives coordinate through the International Nucleotide Sequence Database Collaboration (https://www.insdc.org). Reads are usually stored as compressed archives (e.g. the .SRA format) or in popular formats, particularly FASTQ. The FASTQ format was developed over two decades ago at the Wellcome Trust Sanger Institute (Cock *et al.* 2010) and later popularized by Illumina to store short-read sequencing data. It reports both sequence and per-base quality information, as well as metadata on the sequencing runs in the headers. As data production and storage needs globally scale up, scientific groups have been testing alternative solutions that can provide better data compression, with or without data loss. Popular options include the SAM (Sequence Alignment Map)/BAM (Binary Alignment Map) and CRAM (Compressed Reference-oriented Alignment Map) formats. The SAM format was originally developed as part of the 1000 Genomes Project to store sequence alignments to a reference (Li *et al.* 2009). SAM files can be losslessly compressed to BAM files using BGZF compression, also enabling random lookups through an index file. CRAM was developed in 2011 by the European Bioinformatics Institute with the same purpose of compressing SAM records. The CRAM format specification is maintained by the Global Alliance for Genomics and Health (GA4GH) (Rehm *et al.* 2021). CRAM can, in principle, achieve higher compression by only storing base calls that differ from a reference (Hsi-Yang Fritz *et al.* 2011). Further compression is achieved by storing each SAM column into separate blocks, improving the compression ratio of similar data. CRAM compression can be both lossless and lossy. The latest version, CRAM v3.1, allows an additional 7–15% compression via new custom compression codes that rely on bit-packing and other innovations (Bonfield 2022).

In short-read sequencing, read length is limited by the sequencing by synthesis (SBS) technology (Giani *et al.* 2020), resulting in reads of the same length or nearly the same length (SBS reads may be truncated by quality filters), usually within the 100–500 bp range. In long-read sequencing, sequencing occurs in real time without DNA amplification, imposing significantly fewer constraints on read length. Initially, PacBio sequencing routinely generated reads above 50 kb, with some reads over 100 kb. With the advent of the more accurate Circular Consensus Sequencing (CCS) as the standard sequencing approach and the introduction of PacBio High Fidelity (HiFi) reads in 2019 (Wenger *et al.* 2019), read lengths were confined to a narrower 10–20 kb range, which provides a good compromise between read length and consensus accuracy. ONT is currently the only technology capable of routinely generating reads over 100 kb (ultralong reads; UL) and sometimes above 1 Mbp (whales), but with lower consensus accuracy compared to HiFi.

For both the compressed read data and differences in sequencing approaches mentioned above, assessing read quality is an important factor affecting downstream analyses such as read mapping and genome assembly. Per-base quality is reported by the sequencing platforms using Phred scores (Q scores, or QV for quality values) (Ewing and Green 1998). The formula for the Phred score maps the probability of a particular base being incorrect to an integer Q. The most common formula is the Sanger: $-10\log_{10}(P)$. The Sanger format can encode a Phred quality score from 0 to 93 using ASCII 33 to 126. In real scenarios, Phred scores rarely exceed 60 for reads, but higher scores are possible for individual bases, assemblies, or read maps. Phred scores are encoded in FASTQ, SAM/BAM, or CRAM files. Illumina Phred scores are usually below Q40, while AVITI can reach Q50. In 2014, to reduce storage footprint, Illumina introduced binning for Q scores, in which ranges of Q values are assigned the same Q value (Illumina 2014). By binning the Q values, one can achieve better data compression as more similar values are easier to compress. Originally, PacBio Continuous Long Reads (CLR) had error rates >10–15% (Q8–10). Estimated Q scores for insertions, deletions, and substitutions were stored in different BAM tags. PacBio read quality improved radically with the introduction of CCS and HiFi reads (Wenger *et al.* 2019). HiFi reads are CCS filtered for an average quality of at least Q20, with an average of Q27–30 after filtering. HiFi reads are natively stored in SAM/BAM format and can report significantly higher Q scores (>80) for individual bases in a read. In PacBio’s latest sequencing platform, the Revio, Q scores are also binned to increase storage efficiency and capped at Q40 by default, ranging from Q40 to Q93. Methods to further improve per-base accuracy are continuously being developed, including the popular DeepConsensus approach (Baid *et al.* 2023) that is now integrated in the PacBio sequencing platform, as well as other methods to generate consensus sequences with better calibrated Q scores (Weerakoon *et al.* 2025). ONT reads also had low QV (∼Q10) but have progressively improved over the years. ONT Duplex reads, a special type of library preparation that allows for the reading of both DNA strands which are then merged in a higher-accuracy consensus sequencing, have quality values capped at Q50 when called using the Dorado base caller and are also natively stored in SAM/BAM format (Koren *et al.* 2024).

As more sequencing data becomes available, a single, fast, versatile tool that can compute read summary statistics from a variety of file formats is warranted. As part of gfastar, a tool suite to aid telomere-to-telomere (T2T) genome assembly (Nurk *et al.* 2022, Formenti *et al.* 2022, Rautiainen *et al.* 2023), we have developed and present here rdeval (short for “read evaluation tool”). Rdeval fills a gap in evaluating sequencing reads datasets at scale as it can efficiently compute accurate summary statistics, plot read distributions, and convert between different file formats, facilitating the storage and analysis of large datasets.

## 2 Materials and methods

### 2.1 System and methods

Rdeval works by loading sequencing read files, preprocessing them, computing read statistics, and optionally saving them in different formats. Rdeval v0.0.7 (the version presented hereinafter) accepts all popular sequence read file formats, including FASTA, FASTQ, SAM/BAM, and CRAM, enabling seamless conversion between these file formats. SAM/BAM and CRAM support is provided by HTSLIB (Bonfield *et al.* 2021). Rdeval can process heterogeneous file formats in the same run, e.g. both FASTQ and CRAM can be simultaneously provided without conversion, which also allows combining multiple files in different formats in a single archive. Inputs can optionally be filtered in a preprocessing step to include/exclude sequences based on combinations of criteria. In particular, reads can be filtered by length, e.g. to remove very short reads or to retain only UL reads, and/or they can be filtered by sequence quality to remove low-quality reads or retain only highly accurate reads. Rdeval computes per-read metrics, including read counts and length, average base quality, base composition, and GC content. Rdeval further allows homopolymer compression, a sequence preprocessing step to shrink homopolymer runs to a defined length (usually 1 bp), in linear time without requiring auxiliary space, a feature increasingly useful when dealing with long reads.

**Figure 1.**
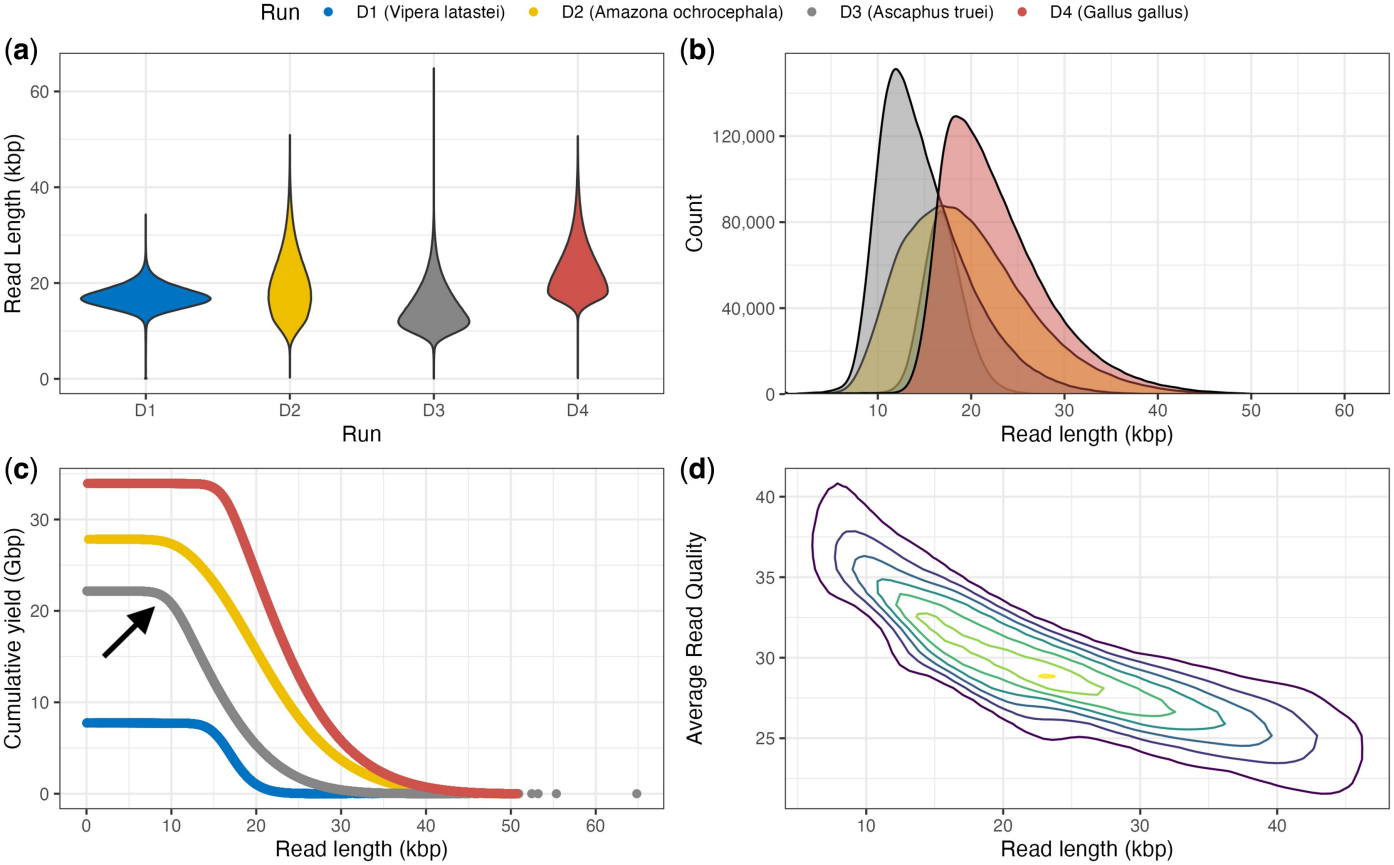
Representative plots from rdeval report using four HiFi sequencing runs from four species (D1: *Vipera latastei*, D2: *Amazona ochrocephala*, D3: *Ascaphus truei*, and D4: *Gallus gallus*). (a) Read length violin plots. D1 shows a tighter read length distribution, and D3 is skewed toward shorter read lengths. (b) Read length density plots. Similar to the violin plots, D3 has shorter reads on average, and D4 has the longest read lengths. (c) Read length inverse cumulative distributions. The distribution can be used to assess read coverage at certain read length cutoffs. For instance, in D3, we can observe that there is about 20 Gbp of coverage for reads 10 kb or longer (black arrow). (d) Read length versus Average read quality, plotted as a 2D contour plot that highlights data density for the four runs combined. The plot shows that read length correlates inversely with average read quality at varying magnitudes, with the highest density of reads in the 20–25 kb read length range and Q25–30 read quality range.

Many features of rdeval are not available in other tools for sequencing data analysis (Table 1, available as supplementary data at *Bioinformatics* online), and these features can be optionally post-processed for downstream analyses, including for data visualization. Key to this process is the generation of a read ‘snapshot’ that stores sorted lengths and Q scores for all reads. The snapshot is a binary .rd file, which stores the information needed for downstream analyses (see the Algorithm section for further details). Binary data is further compressed with gzip. Snapshots maintain information on the original data despite achieving a remarkable reduction in file size compared to regular statistical summarization. The .rd file generated by rdeval can then be used for plotting static and interactive read summaries for one or more files in HTML or PDF reports via a bundled R script. A simple bash wrapper script is provided for convenience to run the R commands. These R commands can be used to generate graphical outputs, including violin plots and histograms illustrating read length, inverse cumulative read length distribution, and the relationship between read length and read quality (Fig. 1). We generated snapshot files and graphical sequence reports for all VGP read data in SRA, and made them available in GenomeArk (https://genomeark.s3.amazonaws.com/index.html? prefix=downstream_analyses/SRA/2025-05-01/). GenomeArk will host rdeval’s snapshots and sequence reports for all sequence data made available in the repository moving forward. Rdeval also computes md5sum information for the original files using OpenSSL (Ooms 2023) and stores it in the .rd files. Md5sum information can be used to uniquely assign summary statistics to the original files from which they were derived, resolving any ambiguities. Additionally, rdeval can be used to randomly downsample reads through the ‘sample’ option, which allows for the extraction of a specific fraction of reads to create subsampled datasets. For optimization purposes, rdeval is coded in C/C++, taking full advantage of object-oriented programming. The code is highly commented, and a verbose option is available for debugging. Expensive operations are multithreaded to minimize runtime overhead, including file decompression and compression during I/O.

All runtime tests presented below were run on a server using 4 cores and 30 GB of memory allocated. Plots were generated using 84 different sequencing datasets from the VGP project, considering both Illumina (*n* = 37) and PacBio (*n* = 47) sequencing platforms (Table 2, available as supplementary data at *Bioinformatics* online). For Illumina, only R1 files were used. The latest CRAM v3.1 was used in the comparisons. To assess quality and read length, downsampled human datasets from CHM13 and HG002 projects were used (Table 3, available as supplementary data at *Bioinformatics* online), AVITI data from the Human Pangenome Reference Consortium (human-pangenomics). The same datasets were also used to evaluate the .rd file size following downsampling. To allow comparison across technologies, each dataset was downsampled to 11 Gbp. A representative report was generated using data from the VGP, specifically PacBio HiFi read files from four species (Table 4, available as supplementary data at *Bioinformatics* online): *Gallus gallus* (SRR30304940), *Vipera latastei* (SRR25383704), *Ascaphus truei* (SRR30223073), and *Amazona ochrocephala* (SRR29949495). Files were downloaded from NCBI SRA and converted to FASTQ, respectively using the *prefetch* and *fasterq-dump* commands from the SRA toolkit. Binary .rd files were generated for each run to store read length, quality values, and base counts. In order to read the binary .rd files into R, a binary interface was written in R using the *bit64* package. Reports are generated from R Markdown directly from the .rd files using the binary interface. Rdeval snapshots for VGP long-read sequencing data were computed by downloading the reads from SRA with *fasterq-dump*. Reads were downloaded and snapshots were computed in just 2 days on a single 32-core machine, and only a few hours when using more than 10 nodes in an high-performance computing environment (HPC). Theoretical size of VGP SRA’s as GZIP FASTQs was computed as 2 bytes per base (sequence and quality) * ∼60Tb considering a gzip compression factor of ∼2.5× (headers were ignored). Data visualization was achieved using the bash command Rscript. A bash wrapper script, generate_report.sh, is provided for convenience to run the R commands, allowing users to toggle static or interactive plotting and output format (HTML and PDF for static plots only). The plots are generated directly from the rdeval .rd files in R, rendered either as static images using the *ggplot2* and *ggMarginal* packages, or interactive plots using the *plotly* package, and the report is rendered using the *render* function from the rmarkdown package. PDF rendering requires installation of a LaTeX distribution such as TinyTeX. For a thorough analysis of all datasets produced under the VGP umbrella, we examined all SRA sample accessions (ERS, SRS) updated as of January 24, 2025. Some accessions were excluded due to mislabeling or ambiguity regarding whether the data were generated using the Sequel, Sequel II, or Revio platform or cases where sequencing data of a specific biosample were produced using different sequencing instruments (Table 5, available as supplementary data at *Bioinformatics* online). In the coverage vs. contiguity analysis, regression lines were computed excluding outliers. Coverage was computed by dividing the total base pair sequences with PacBio HiFi by the assembly size of the primary assembly. All scripts used for figures and analyses in the manuscript are available here: https://github.com/vgl-hub/rdeval-manuscript.

### 2.2 Algorithm

**Figure 2.**
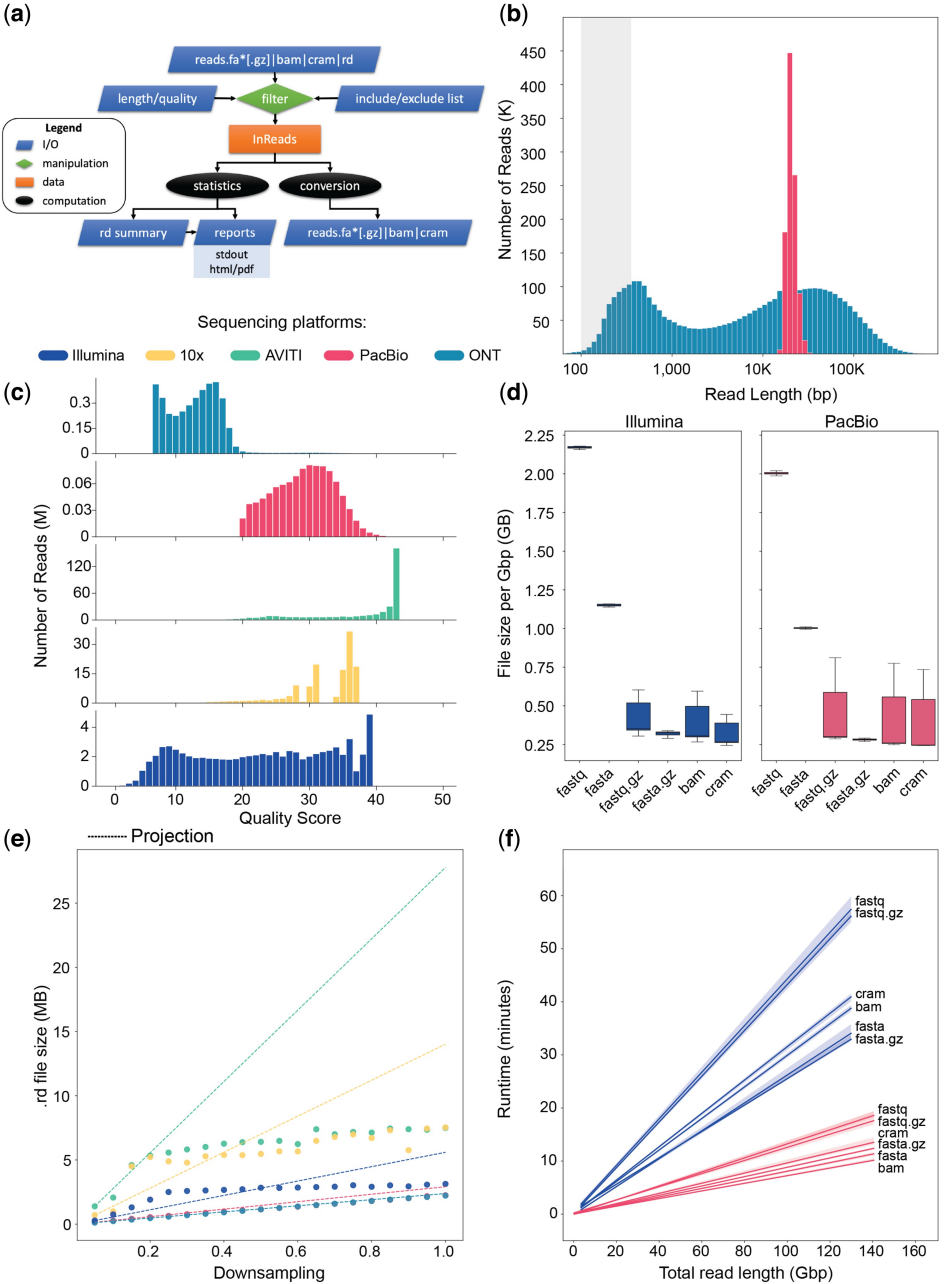
Sequencing read evaluation with rdeval. (a) Schematic of rdeval workflow. Inputs include genome assemblies in fasta, fa*[.gz], BAM, CRAM formats, and include/exclude lists as bed coordinate files for filtering. (b) Length distribution of reads from two representative human genomic datasets (CHM13 and HG002). (c) Quality distribution for the same data sets. Note that PacBio HiFi reads are at least Q20 and are capped at Q40. (d) Comparison of compression levels of 80 sequencing datasets (datasets under 1 Gbp in total read length were excluded) included in the VGP project across different file types (FASTA [.GZ], FASTQ [.GZ], BAM, and CRAM) and sequencing platforms (Illumina and PacBio; Table 2, available as supplementary data at *Bioinformatics* online). (e) Relationship between original file size and.rd file size in different data sets: the *X*-axis represents downsampling, while the *Y*-axis shows.rd file sizes in MB. The test has been performed on five different sequencing runs from human genomic data sets (Table 3, available as supplementary data at *Bioinformatics* online) generated by different sequencing platforms. All data sets were initially downsampled to 11 Gbp, corresponding to the downsampling fraction of 1. Each downsampled dataset was further downsampled to generate the remaining data points. The.rd size at the first downsampling level (0.05) has been used to estimate theoretical projections at subsequent steps. Projections are shown in dashed lines. (f) Runtime (minutes) versus data set size (Gbp) for 84 sequencing data sets from Illumina (*n* = 37) and PacBio (*n* = 47) platforms across various file formats.

A schematic outline of rdeval’s general mode of operation is presented in Fig. 2a. In rdeval, input is processed in parallel read-by-read, building a data structure containing read name (optionally), quality, length, and base composition, and then the data are summarized at the end. In case of file conversion or read filtering, outputs are streamed to disk, minimizing memory footprint. Rdeval optional read snapshots, ie highly condensed representations of relevant read features that can then be stored in rdeval’s own highly compressed .rd files. An .rd file is currently made of a binary header followed by a gzip-compressed binary data section. The header contains information on the files that went into the generation of the snapshot file, along with their md5sum values. The header is followed by a binary uint64 with the size of the uncompressed data. Data follows as gzip-compressed binary data. Binary data stores aggregated statistics, including read length distributions, per-read quality scores (mean and median), and base composition summaries. Different int types (8, 16, 64) help compression under different scenarios of read length distribution, and refer to specialized data structures to store read length/quality pairs. Snapshots’ format specification is further detailed in rdeval’s ReadTheDocs documentation (https://rdeval-documentation.readthedocs.io). Also in the ReadTheDocs documentation are examples of how to read .rd files in C/C++ and R. Critically, rdeval’s .rd data format allows extensive parallelization of the process by generating tiny intermediate files, one for each read input file, that can then be combined in a few seconds. Once .rd files are generated, plotting is rapid (on average 1m35s for interactive plots, 7 s for static HTML and PDF reports, based on generating reports from 221 SRA runs) and can be repeated for multiple samples with minimal computational overhead (also in rdeval’s ReadTheDocs documentation). We note that the .rd file format does not allow reconstruction of the raw data, and that gzip is used only to reduce file size further.

### 2.3 Implementation

We conducted a series of validation tests and analyses for rdeval’s ability to evaluate read length and quality. In two representative human datasets from T2T-CHM13 and HG002 (Table 3, available as supplementary data at *Bioinformatics* online), rdeval correctly identified short-read technologies read lengths ranging from 100 to 500 bp. HiFi reads showed a unimodal distribution centered at 20 kb with a low standard deviation (Fig. 2b). The ONT UL dataset (Logsdon *et al.* 2022) showed a dispersed bimodal distribution of read lengths, with peaks at 500 bp and 40 kb (Fig. 2b); this dataset was generated with Rapid Sequencing Kit (SQK-RAD004) following modification to the manufacturer’s protocol and sequenced on a GridION using a FLO-MINI106 R9.4.1 flow cell and MinKNOW (v.2.0–v1.0.12). In terms of quality, Illumina data show a wide range of quality scores (Q2–39), with the mode at Q39. 10x Genomics linked reads and AVITI reads by Element Biosciences present qualities spanning Q16–43 with high-frequency at Q36 and Q43, respectively. HiFi reads showed quality scores in the range of Q20–40, with the highest frequency at Q30. ONT UL reads were mostly below Q20, showing a bimodal distribution centered at Q7 and Q17 (Fig. 2c). Additionally, we observed a small subset of reads shorter than 1 kb close to Q90, possibly due to a base-calling artifact.

When comparing different file formats, considering 1 Gbp of data per sample and excluding samples with smaller input sizes, we observed that unaligned BAM and CRAM together achieved better compression over plain text and compressed FASTQ files by 6.4-fold and 1.2-fold on average, respectively. Furthermore, the variability observed in BAM/CRAM and FASTQ.gz file sizes was largely influenced by differences in their compression processes, which are in turn affected by the variability in base quality scores (Fig. 2d). CRAM could be considered as the format of choice when high compression efficiency is needed, especially for large repositories, as it achieves the smallest sizes while maintaining essential information, including sequence and base call quality. Interestingly, after homopolymer compression, read datasets were usually on average 29.5% smaller than their uncompressed counterparts (Fig. 1, available as supplementary data at *Bioinformatics* online). We also investigated the relationship between the original file size and the .rd file size. The file size ratios compared to the original file range from 9000-fold for long-read datasets to 650-fold for short-read datasets on average, essentially removing any overhead when sequence data metrics are needed for data analytics. Our tests show that the .rd file size increases sublinearly with increasing sampling fraction across all samples, improving file size reduction gains over the original files as more data is processed (Fig. 2e). This can be expected, since read length and quality variation will quickly saturate, and measurements on reads with the same length and quality can be efficiently gzip-compressed. For instance, in the case of the AVITI dataset, we observed a reduction in file size of approximately 75% when comparing the expected size to the empirical one.

Rdeval can compute metrics in O(N) time with runtimes of approximately 5–10 min for a typical 30× coverage human dataset input using long-read data (PacBio HiFi or ONT), and about 30 minutes for Illumina short reads. Runtime variability depends on both the sequencing platform and the file format. In our experiment with datasets up to 150 Gbp, all runs took <1 h (Fig. 2f). Illumina datasets consistently exhibit higher runtimes than PacBio datasets of equivalent sizes, which is due to the need to process many more reads of shorter lengths. BAM achieves overall runtime best performances for both sequencing platforms while preserving all the information in the datasets. The maximum memory footprint recorded was ∼10 GB, observed in only a few FASTA datasets exceeding 100 Gbp. On average, however, rdeval has modest RAM requirements (<4 GB).

**Figure 3.**
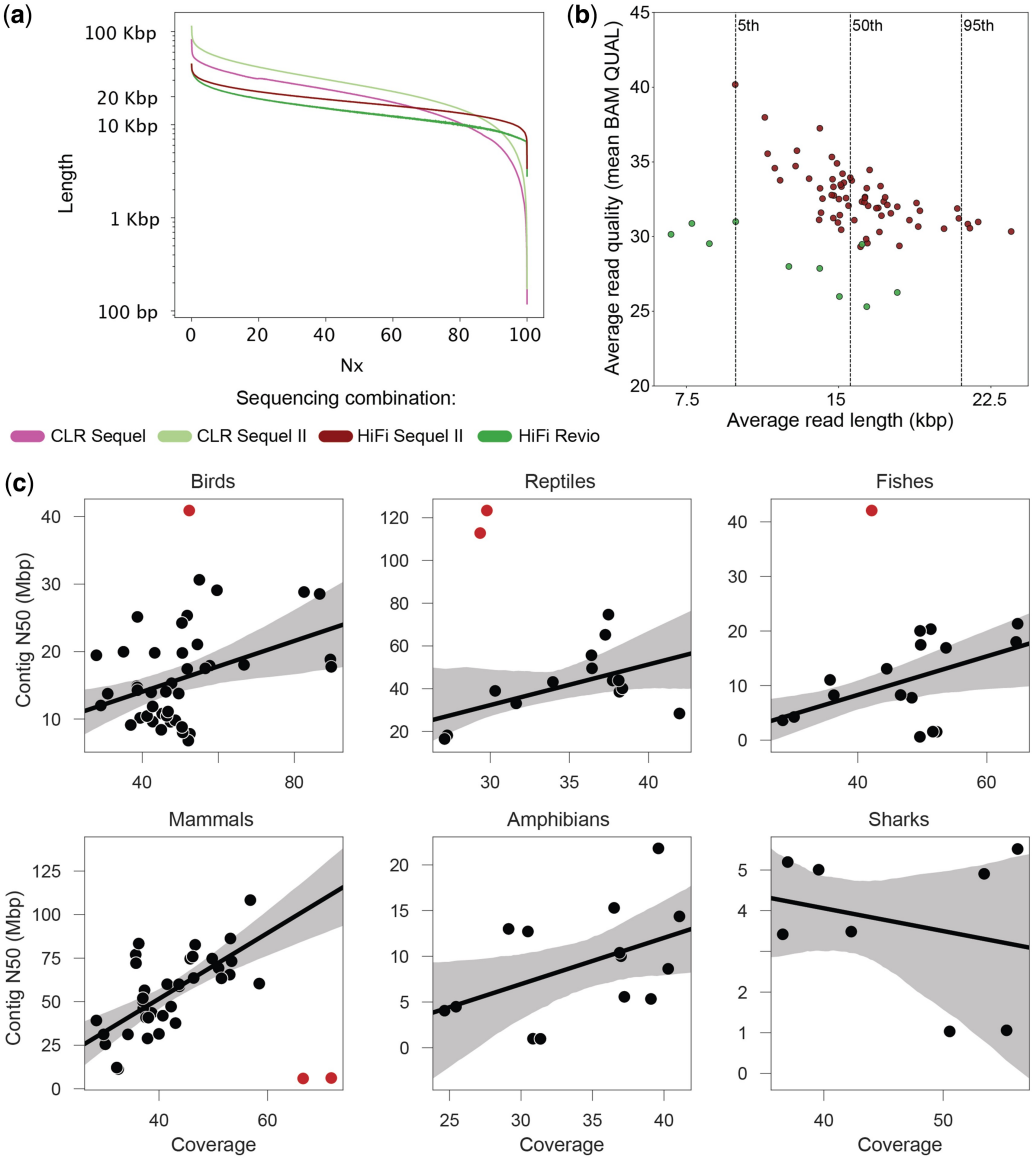
(a) Average read length Nx plot using all long-read VGP datasets (Table 5, available as supplementary data at *Bioinformatics* online). Most reads were shorter than 100 kb on the Sequel Instrument. Read lengths consistently improved with Sequel II. HiFi consensus read lengths were longer on the Sequel II, and shorter with the Revio, likely as a consequence of shorter movie times. (b) Average read length versus average read quality across VGP datasets and instruments. Average read quality is calculated as the mean of the BAM QUAL field. Note that the rq tag in the BAM file represents the estimated “read quality” from Revio and Sequel II, and on Revio, rq may be higher than mean (QUAL) in the final output. Datasets are color-grouped by sequencing platform. Datasets generated by mixed sequencing instruments were excluded from the analysis. Q scores are not available for CLR reads downloaded as FASTQ from SRA. HiFi data show an inverse relationship between read length and quality, consistent with fewer number of passes available for consensus in longer reads. The difference observed between Sequel II and Revio data is only a representational change, due to capping of Q scores in Revio instruments. (c) Correlation between PacBio HiFi sequencing coverage and contig N50 in VGP genomes. Outliers are highlighted.

Snapshots can be used to interactively display the datasets, generating complex data visualizations, including by re-analyzing read length and quality distribution without going back to the original data, which is particularly important in the case of large datasets. This is a useful feature, particularly when analyzing several datasets together or in consortia hosting hundreds or thousands of datasets in dedicated repositories. Interactive visualizations display summary metrics, allow toggling which datasets to display, zooming, visualizing individual values and data points, and exporting the new plots as PNG files. To showcase this functionality, we computed rdeval snapshots for 3,010 individual long-read sequencing runs, ie most of VGP's data with SRA accessions to date. While they represent 63.5 Tbp from 4.4 billion sequencing reads (roughly 50 TB if stored as GZIP-compressed FASTQs), the total file size of the snapshots is just 9.5 GB. These snapshots can be used to analyze the evolution of sequencing technologies over time. For instance, variations in read length can be observed across VGP datasets generated using different platforms and technologies. The average PacBio read length is 10 803 bp for CLR data generated with a Sequel instrument, 15 599 bp for CLR data produced using the Sequel II platform, 16 116 bp for Sequel II HiFi data, and 12 516 bp for Revio data, respectively (Fig. 3a). Quality scores have also changed significantly and tend to correlate inversely with read length, though caution should be exercised when interpreting Q scores, distinguishing between actual changes in accuracy and representational changes due to binning, capping, or other factors (Fig. 3b).

Snapshots and reports can be useful to investigate the genomic resources. For instance, we analyzed the correlation between PacBio HiFi coverage and contig N50 in VGP genomes. We observed as expected that further increasing coverage leads to more contiguous assemblies, but there were clear relationship differences in different clades (Fig. 3c). This correlation was evident in all clades except sharks, which have unusually repetitive genomes for vertebrates, and where clade-specific differences in repeat content may explain the result. Six interesting outliers were noticeable. In birds, the king vulture (*Sarcoramphus papa*) was an outlier with a contig N50 of 43.2 Mbp. The king vulture appears to have particularly large macrochromosomes compared to other birds, which can explain the high N50 contig. Similarly, in reptiles, the rock iguana (*Cyclura pinguis*) showed exceptionally high contig N50 values (112.8 Mbp). This is not totally unexpected owing to the peculiar chromosome structure of the genome, with the three largest chromosomes making up half the genome size and the smallest being ∼300 Mbp. Iguanas have been reported to differ in the accumulation and distribution of interstitial telomeric sequences, which suggests chromosomal fusions in some species (Altmanová *et al.* 2016). In fish, the coelacanth (*Latimeria chalumnae*) also showed a high contig N50 (42.0 Mbp), which is likely explained by its unusual genome size (2.9 Gbp) and content compared to non-lobe-finned fish (genome size ∼1 Gbp on average). In mammals, the Botta’s pocket gopher (*Thomomys bottae*) showed very low N50 values (5.9 Mbp for haplotype 1 and 6.2 Mbp for haplotype 2) compared to other mammals despite relatively high sequencing coverage (134.8 Gbp). Genetic studies in gophers have shown high levels of chromosomal variability across and within populations, with varying chromosome size and numbers, and generally higher than usual repeat content for mammals (Voss *et al.* 2025). This relatively high proportion of repetitive DNA is likely to explain the observed results.

## 3 Discussion

Using rdeval, we were able to highlight interesting features in existing read datasets, including the marked differences in read length and quality distribution as a consequence of the rapid evolution of DNA sequencing technologies. For PacBio long-read sequencing, the major transition was from CLR generated by the Sequel platform to HiFi on the Sequel II sequencing platform. The introduction of the Revio platform coincides with a reduction in average read length. Interestingly, our analysis of homopolymer compression, a widely used approach in genome assembly to overcome the challenges posed by inaccurate base calling of homopolymers in long-read sequencing platforms (Bankevich *et al.* 2022, Rautiainen *et al.* 2023), highlights that this strategy results in significant data compression, potentially removing useful information in short mononucleotide sequences and satellite DNA homopolymers.

Rdeval also showcases how to improve efficiency in large sequencing projects and large public repositories, by choosing more efficient file formats for the storage of raw sequencing reads. BAM and CRAM are valid alternatives to FASTQ. BAM is a good compromise between compression and data retrieval, particularly when dealing with long reads, whereas CRAM achieves the best compression. Rdeval natively supports these file formats and is ready to be integrated into production workflows to assess and compare the quality of sequencing runs. Rdeval can also become a standard component of large sequencing projects and repositories to ease access to key read quality metrics.

Compared to established tools for sequencing data analysis, rdeval possesses a combination of desirable features not available in similar tools. For instance, FastQC (Andrews 2010) excels at assessing biases within short-read data, such as duplicated sequences and overrepresented k-mers. However, it lacks input manipulation options and generates per-base aggregated metrics incompatible with long-read sequencing. Other popular tools like SeqKit (Shen *et al.* 2016) and seqtk (Li 2012) have additional functionalities (e.g. read length quartiles, CpG counts, etc.), but lack support for BAM/SAM available in rdeval and FastQC. Notably, rdeval shows high scalability potential due to multithreaded processing, a feature lacking in FastQC and seqtk. Rdeval offers unique features like CRAM support, coverage calculation, and read snapshotting in highly compressed binary outputs, which are ideal for analyzing large, mixed-format sequencing datasets. While rdeval is optimized for WGS data and is particularly efficient in the context of long reads, it can also be used for RNA-seq, amplicon sequencing, and microbial genomics. Sequence content will not have any discernible impact on rdeval’s efficiency.

We will continue to add functionalities to rdeval. For example, a ‘quick’ mode that can generate approximate summary statistics by skimming through the data quickly until convergence. We will also evaluate whether to include the functionalities from other tools presented in our comparison table and currently missing from rdeval, to provide all useful sequence manipulation functionalities in a single tool. We will also continue to improve the efficiency of the tool, particularly by decreasing its runtime and reducing snapshot file size. Examples of future improvements are faster I/O operations and the use of hashing techniques to represent high-frequency reads with identical properties, particularly abundant in short-read datasets, to further reduce snapshot file size. Rdeval also does not currently analyze paired-end reads as pairs, though read pairs can be easily handled separately and compared. Another direction of development will involve testing and integrating more specialized and efficient compression algorithms for genomic sequences such as CoLoRd (Kokot *et al.* 2022) or JARVIS3 (Sousa *et al.* 2024).

## Supplementary Material

btaf416_Supplementary_Data

## Data Availability

No new data were generated in support of this research. VGP data are publicly available under NCBI BioProject PRJNA489243.
