# Supplementary material for: Evaluation of sequencing reads at scale using rdeval
Source: Bioinformatics. 2025 Jul 22;41(9):btaf416. doi: 10.1093/bioinformatics/btaf416 (PMC12401588; doi:10.1093/bioinformatics/btaf416)
Supplement: btaf416_Supplementary_Data [file btaf416_supplementary_data.zip › btaf416_Supplementary_Data/captions[AU].docx]

**Supplementary Figure 1**. Original file size vs. homopolymer-compressed size for data sets of **Supplementary Table 2**.

**Supplementary Table 1:** Comparison of features and operations computed by rdeval against Seqkit (Shen *et al.*, 2016), seqtk (Li), and FastQC (Babraham Bioinformatics - FastQC A Quality Control tool for High Throughput Sequence Data). Various metrics, supported formats, and statistics for sequencing data sets are missed in alternative tools.

**Supplementary Table 2:** Sequencing data sets from the VGP project used for testing the runtimes, homopolymer-compression, and level of compression of different formats (FASTA [.GZ], FASTQ [.GZ], BAM, and CRAM).

**Supplementary Table 3:** Representative human sequencing data sets used in the study.

**Supplementary Table 4:** Sequencing data sets from NCBI SRA used to generate representative plots in **Figure 1**.

**Supplementary Table 5:** List of SRA accessions used in **Figure 3a,b.**
