# Supplementary figures and images for: Evaluation of sequencing reads at scale using rdeval
Source: Bioinformatics. 2025 Jul 22;41(9):btaf416. doi: 10.1093/bioinformatics/btaf416 (PMC12401588; doi:10.1093/bioinformatics/btaf416)

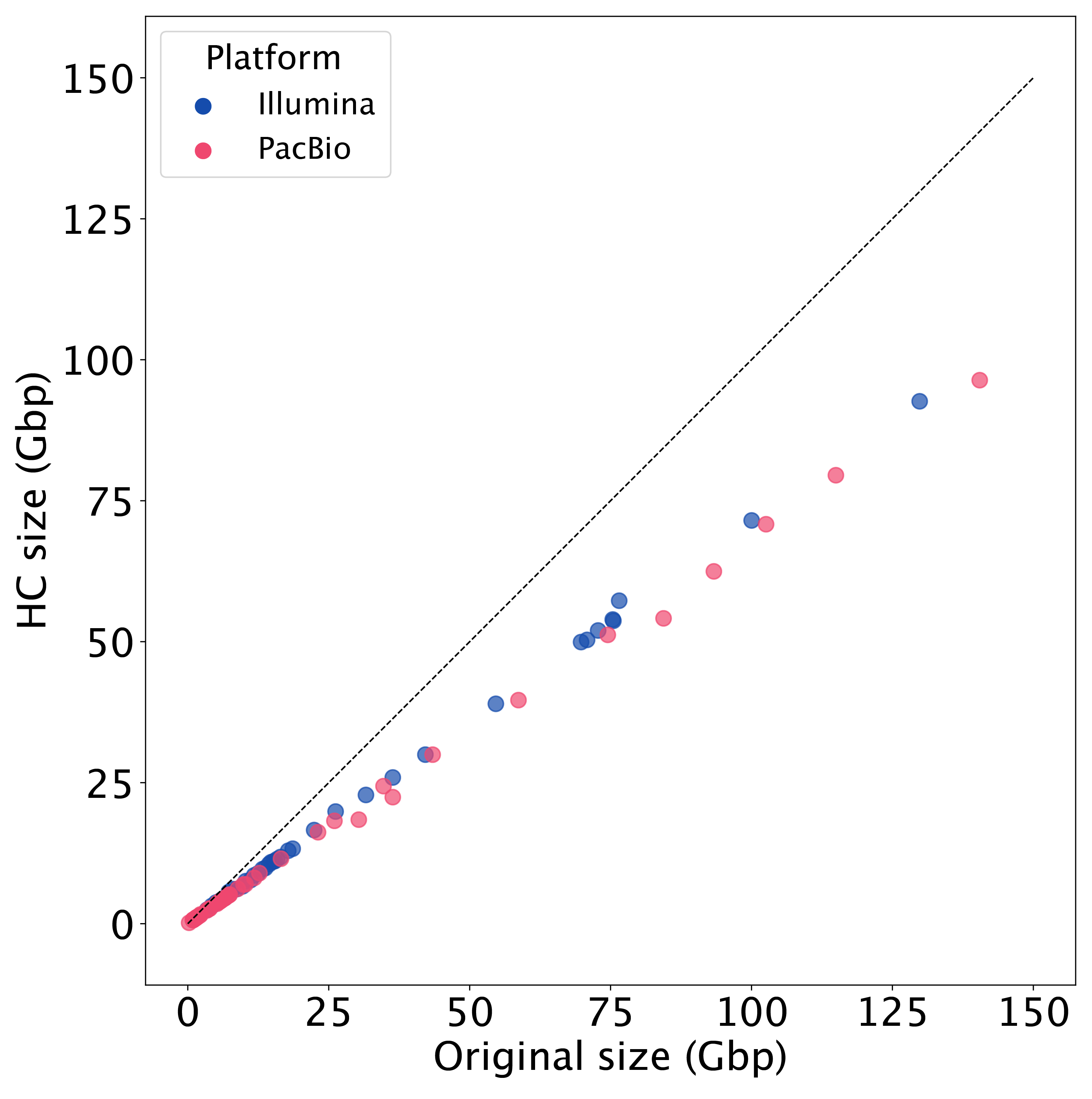

Supplement: btaf416_Supplementary_Data [file btaf416_supplementary_data.zip › btaf416_Supplementary_Data/Supplmentary Figure 1.png]
